# A comparative study of the effectiveness of group-based cognitive behavioral therapy and dialectical behavioral therapy in reducing depressive symptoms in Iranian women substance abusers

**DOI:** 10.1186/s41155-018-0094-z

**Published:** 2018-06-25

**Authors:** Sara Sahranavard, Mohammad Reza Miri

**Affiliations:** Social Determinants of Health Research Center, Faculty of Public Health, Birjand University of Medical Sciences, Birjand, Iran

**Keywords:** Cognitive behavioral therapy (CBT), Dialectical behavioral therapy (DBT), Depressive symptoms, Substance abusers

## Abstract

Various therapeutic approaches have been used to improve depressive symptoms in substance abusers. In a quasi-experimental study with a pretest-posttest design and experimental and control groups, we examined and compared the effectiveness of two group-based treatment strategies—cognitive behavioral therapy (CBT) and dialectical behavioral therapy (DBT)—in reducing depressive symptoms among Iranian women substance abusers. The statistical population included all female addict patients who referred to addiction treatment centers of Birjand city in 2015. A sample of 30 subjects were selected through the available sampling method and randomly assigned into experimental (CBT and DBT) and control groups (each group, 10 patients). The data collection instrument was the Beck Depression Inventory (BDI) questionnaire. The patients in the experimental groups were given skills in eight sessions of 90 min. The data were analyzed by the SPSS-19 software by using mean, standard deviation, and percentages at the descriptive level and analysis of covariance (ANCOVA) test at the inferential level. The comparison of the mean depression score before intervention in all the groups showed no significant difference. However, after intervention, the findings showed that both CBT and DBT interventions could reduce the mean scores of depression in women substance abusers, 17.5 ± 3.0 vs 29.3 ± 4.1 (*F*[1,17] = 51.91, *p* value < 0.01) and 14.7 ± 1.8 vs 29.3 ± 4.1 (*F*[1,17] = 106.62, *p* value < 0.01), respectively, for CBT and DBT. Post-treatment effect sizes were large and did not differ statistically for CBT (*η*_p_^2^, 0.75) and DBT (*η*_p_^2^, 0.86). Therefore, this study highlights the importance of CBT and DBT skills training to substance abusers and provides initial evidence of their effectiveness.

## Background

Drug dependence is a chronic reversible disorder with profound social, psychological, physical, and economic effects. It leads to personality destruction and heavy costs on individuals. Many theories have proposed that the etiology of addiction has a significant diversity and complexity. Although some researchers focus genetic factors and environmental stress as the main aspects of substance abuse (Bevilacqua & Goldman, 2009; Enoch, 2012; Kendler, Gardner, & Prescott, 1997; Sinha, 2009; Wong & Schumann, 2008), some others emphasize sociological and psychological aspects of the disease (Adrian, 2003; Kerridge, 2009). The World Health Organization’s (WHO) annual report shows that there are about 200 million opiate addicts in the world. More interestingly, the highest prevalence of opiate addiction has been reported in Iran (2.8%), followed by Kazakhstan (2.3%) and Belarus (1.2%) (United Nations Office on Drugs and Crime (UNODC), 2005). Recent studies have found that high consumption of addictive substances such as alcohol and opium can cause a wide range of cognitive disorders as well as deficiencies in learning, memory, information processing, executive functions, verbal and spatial abilities, problem solving, and visual ability (Kosten & George, 2002; Prosser et al., 2006).

Psychiatric disorders along with substance abuse disorders have had devastating effects on physical, psychological, social, and family health. Basic depression, anxiety, borderline personality disorder, and antisocial personality disorder are the most common psychiatric diagnoses among addicts (Astals et al., 2009; Roberts, Roberts, & Xine, 2007). Beck’s theory of depression (Beck, Rush, Shaw, & Emery, 1979) proposes that difficult early life experiences can make an individual vulnerable to depression through the development of unhelpful core beliefs about the self, others, and the world. These unhelpful core beliefs can then be activated later in life, triggered by stressful events. An episode of depression affects the processing of information, leading to biased and negative interpretation of interpersonal experiences, with an increased sense of isolation and reduced levels of activity (Grant, Mills, Mulhern, & Short, 2004; Leahy, 2017). Meanwhile, patients who suffer from addiction and depression are more likely to be seen in psychiatric sanatorium or addiction treatment centers than the general population. These people will need more help than other people having only a specific illness (Fitzsimons, Tuten, Vaidya, & Jones, 2007). Substance abuse can lead to different depressive symptoms such as lack of enjoyment and affection, disappointment, feelings of resignation, forgetfulness, and isolation. As a case example, about one third to half of those who are opioid abusers and also 40% of alcohol abusers exhibit at least one of the diagnostic criteria for major depressive disorders once in their lifetime (Davis, Uezato, Newell, & Frazier, 2008; Nunes & Levin, 2004; Ostacher, 2007). In 2015, Edlund et al. (2015) reported that the rate of depression among drug users is about four times more than others. According to the results of some studies (Dolan, Martin, & Rohsenow, 2008; Fitzsimons et al., 2007; Quello, Brady, & Sonne, 2005), since mood disorders increase vulnerability to drug abuse and addiction, the diagnosis and treatment of the mood disorder can reduce the risk of subsequent drug use and relapse in addicts. It should be noted that depression by creating symptoms such as helplessness is considered as an obstacle for leaving addiction. Besides, an important feature of addiction is the high drug craving that may promote the continuation of consumption (Dolan et al., 2008). Therefore, the diagnosis and treatment of drug use disorders may reduce the risk of developing other mental illnesses and, if they do occur, lessen their severity or make them more amenable to effective treatment. For treating emotional problems such as depression disorder, different therapeutic techniques have been developed including drug therapy and non-drug therapy such as psychotherapy, mindfulness cognitive therapy, cognitive behavioral therapy (CBT), and dialectical behavior therapy (DBT) (Hayes, 2004; Pettinati, O’Brien, & Dundon, 2013).

CBT is a type of psychotherapy that helps patients to dissect the relationships among their emotions, cognitions, and behaviors in order to identify and reframe irrational and self-defeating thoughts, which in turn improves their mood and alters their behaviors (Cukor, Pencille, Rosenthal, & Kimmel, 2017; Haynes, 2015; Hofmann, Asnaani, Vonk, Sawyer, & Fang, 2012). Research and clinical practice have shown CBT to be effective in reducing symptoms and relapse rates in a wide variety of psychiatric disorders (Knapp & Beck, 2008). Churchill et al. (2010) reviewed the effectiveness and acceptability of cognitive behavioral therapies (CBTs) compared with all other psychological therapy approaches for acute depression. According to a research study by Beltman, Voshaar, and Speckens (2010), CBT for depression was more effective than control conditions such as waiting list or no treatment, with a medium effect size. Dutra et al. (2008) reported the efficacy of CBT for cannabis dependence, with evidence for higher efficacy of multi-session CBT versus single session or other briefer interventions and a lower dropout rate compared to control conditions. In another study, the cognitive-behavioral stress management group training was examined as a useful interventional method for addicts who are under methadone maintenance therapy (Jandaghi, Neshat-Doost, Kalantari, & Jabal-Ameli, 2013).

Dialectical behavioral therapy (DBT), along with CBT approach, is another of the methods which can be an effective treatment for depression in patients with substance abuse. DBT includes skills of distress tolerance, emotional regulation skills, and interpersonal relations skills as well as the skills of mindfulness (Valentine, Bankoff, Poulin, Reidler, & Pantalone, 2015). DBT also provides a combination of cognitive-behavioral strategies for the development of cognitive skills and emotional and behavioral effects (Jomphe, 2013). This treatment therapy can increase ability to achieve positive results systematically and also facilitate reduction in maladaptive behaviors through mechanisms on effective coping skills (Burmeister et al., 2014). Lynch, Morse, Mendelson, and Robins (2003) reported that DBT skills training and telephone coaching may offer promise to effectively augment the effects of antidepressant medication in depressed older adults. Several randomized clinical trials have found that DBT for substance abusers decreased substance abuse in patients with borderline personality disorder (Dimeff & Linehan, 2008). According to Karbalaee and Ahadi (2012), DBT can be effective for declining the rate of major depression and reducing suicide tendency.

Given the high prevalence of drug abuse and that substance abuse is one of the most complex problems with different physical, psychological, behavioral, and social aspects and due to the lack of studies comparing the effectiveness of group-based CBT and DBT in the treatment of depression in substance users, the present study aimed to compare the effectiveness of these two treatment strategies in reducing depressive symptoms in Iranian women substance abusers.

## Methods

### Participants

In a quasi-experimental before-after controlled study, all female addict patients who referred to addiction treatment clinics in Birjand in 2015 were selected by availability sampling method. All patients were chosen voluntarily whereas their own satisfaction was provided. The inclusion and exclusion criteria were included in this study.

Inclusion criteria are as follows: (1) female subjects between the ages of 25 and 40 years, (2) having the drug abuse based on the diagnostic criteria of DSM-IV, (3) passing 1 month from successful detoxification, and (4) having the Beck depression score higher than 18.

Exclusion criteria are as follows: (1) having psychotic bipolar disorders, (2) suffering especial physical illness at the time of performing research, and (3) illiteracy at reading and writing.

After application of the exclusion criteria, 20 subjects were excluded from the study. The remaining patients were allocated into the experimental (including CBT (*n* = 10) and DBT (*n* = 10) groups) and the control (*n* = 10) groups by using simple random sampling (SRS) method. Measurements were taken before and after the group-based intervention period, and this is known as pretest-posttest design.

### Instruments

So far, common screening instruments have been used for quantifying levels of depression such as Beck Depression Inventory (BDI), Zung Self-Rating Depression Scale (SDS), Major Depression Inventory (MDI), and Hamilton Depression Rating Scale (often abbreviated to HAM-D, HRSD, or HDRS). (Bech, Rasmussen, Olsen, Noerholm, & Abildgaard, 2001; Beck, Steer, & Brown, 1996; Hamilton, 1960; McDowell, 2006; Zung, 1965). The BDI is the most widely used self-rating instrument for measuring depression severity (Wang & Gorenstein, 2013). The reliability of the Beck questionnaire has been estimated to be appropriate with the mean Cronbach’s alpha value of 0.86 (ranged 0.73 to 0.92) (Stockings et al., 2015). In addition, according to the survey by Ghassemzadeh, Mojtabai, Karamghadiri, & Ebrahimkhani, 2005, the BDI-II-Persian had high internal consistency (Cronbach’s alpha = 0.87) and acceptable test-retest reliability (*r* = 0.74). It is worth mentioning that the BDI test consists of 21 groups of statements scored upon the 4-point Likert scale, ranging from 0 to 3 with the total score ranged 0 to 63. Higher total scores indicate more severe depressive symptoms. Depression was categorized as minimal depression (score 0 to 9), mild depression (score 10 to 18), moderate depression (score 19 to 29), and severe depression (score 30 to 63).

### Procedure

The study was approved by the Research Ethics Committee of the Birjand University of Medical Sciences (Birjand, Iran). All patients signed a consent form prior to participating in the study. The consent form is attached to the beginning of the survey that informed them about the study and assured them that their responses would only be used anonymously for research purposes on a voluntary basis. After informing the patients about the study’s purpose, a paper-and-pencil survey was administered to the patients who had volunteered to participate in the study during course hours. This survey was created using demographic variable items and study instruments as described in the “Instruments” section.

### Intervention

The patients in the experimental group (CBT and DBT) were given skills in eight 90-min sessions. The content of the CBT and DBT training courses are respectively listed in Tables 1 and 2. During this time, the control group did not receive any training and was on the waiting list. One week after intervention training, all individuals in the experimental and control groups completed the Beck Depression Inventory (BDI).

**Table 1:** The content of CBT training courses

Training courses	Session
Welcoming, an overview of the structure of the meetings, laws and the importance of doing it, raised issues in relation to depression, provide information to members about depression, relaxation and cognitive logic, obtaining feedback	1
Educating members about reality and perception, consciousness and the difference between feelings and thoughts, providing homework, getting feedback	2
Review assignments, the second stage of progressive relaxation training to get members thinking about “why they are upset,” training to members on the five cognitive error, providing homework, getting feedback	3
Review assignments, the third stage of progressive relaxation training, training to members on other cognitive errors, learning the techniques of distraction, focusing on an object, providing homework, getting feedback	4
Browsing assignments, the fourth stage of progressive relaxation training, discussion about recent emotional experiences, imagination and role play, sensory awareness training, mental training, providing homework, getting feedback	5
Browsing assignments; discussion about emotional experiences and techniques learned from the past session; acquainting the members with recognition of the fundamental words such as love, success, and perfectionism; training of memory techniques and imagination; providing homework; getting feedback	6
Browsing assignments, discussion about dos and don’ts as one of identification techniques of fundamental schema, investigating the role of cognitive distortions in the creation and persistence of depression, learning techniques to count opinions, delivering assignments, getting feedback	7
Review assignments, summing up the meeting by the therapist and members in the group, donating gifts	8

**Table 2:** The content of DBT training courses

Training courses	Session
Mindfulness: Wise mind, “What” and “How” skills. Implement wise mind techniques, specific acceptance skills, and conscious communication with others. Its main purpose is to create an individual’s ability to control attention through non-judgmental teaching style and being efficient	1,2
Interpersonal Effectiveness: Myths, Priorities, DEAR MAN, GIVE and FAST skills. Teaching of distraction techniques from the self-harming behaviors and thoughts by doing pleasurable activities such as participating in doing tasks, comparing yourself with those who have a superior status, creation of deliberate positive emotions, suppression of painful conditions, and substituting the thoughts in order to increase the tolerance power of distress and suffering. Teaching emotional identification techniques, overcoming obstacles of healthy emotions, and confrontation with emotions in which one is taught how to calm down himself/herself and can control and adjust his/her emotions.	3,4
Emotion Regulation: Check the Facts and Opposite Action. Teaching conscious attention techniques and interpersonal key skills and courageously listening in order to become more socialized and repairing the person’s relationship with others.	5,6
Distress Tolerance: Introduction to Crisis Survival Skills, and ACCEPTS, Self-Soothe and IMPROVE the Moment, TIP Skills for Managing Extreme Emotions, Pros and Cons, Introduction to Reality Acceptance Skills, and Radical Acceptance, Turning the Mind and Willingness, Mindfulness of current Thoughts.	7,8

The therapists in this study have master’s degree level of education in psychology and have all specialized expertise in the cognitive and dialectical behavioral therapies. Besides, they had more than 10 years of experience in dealing with addicts and substance abusers and have also passed the supplementary training in this regard.

### Data analysis

All statistical analysis was performed using the SPSS software, version 19 (SPSS Inc., Chicago, Illinois). Results were expressed as mean ± standard deviation (SD) for quantitative variables and percentages for categorical variables. The analysis of covariance (ANCOVA) test was also used to assess the differences between the mean scores of depression in both the control and experimental groups in order to demonstrate the effects of skills training strategies on depressive symptoms in women substance abusers. ANCOVA is a combination of an analysis of variance (ANOVA) and a regression analysis that adjusts the mean scores for each treatment group by that of the mean deviation for the covariate (i.e., the depression scores before intervention). The assumptions of ANCOVA are similar to those of ANOVA (normality and equal variance of residuals) with an additional assumption, homogeneity of regression slopes. Kolmogorov–Smirnov and Levene’s *F* test, respectively, were used to evaluate the assumptions of normality of the variables and equality of variances. Statistical significance was determined as a *p* value of ≤ 0.05.

## Results

The mean age of study subjects in this study was 34.1 years (range 25–40 years) and all were married. More than half (63.3%) of the patients were illiterate and nine (30%) under diploma, and 6.7% had diploma. The most frequently used drug was opium (56.9%) followed by tobacco (27.4%) and cannabis (15.7%). All patients had at least one relapse.

**Fig. 1:**
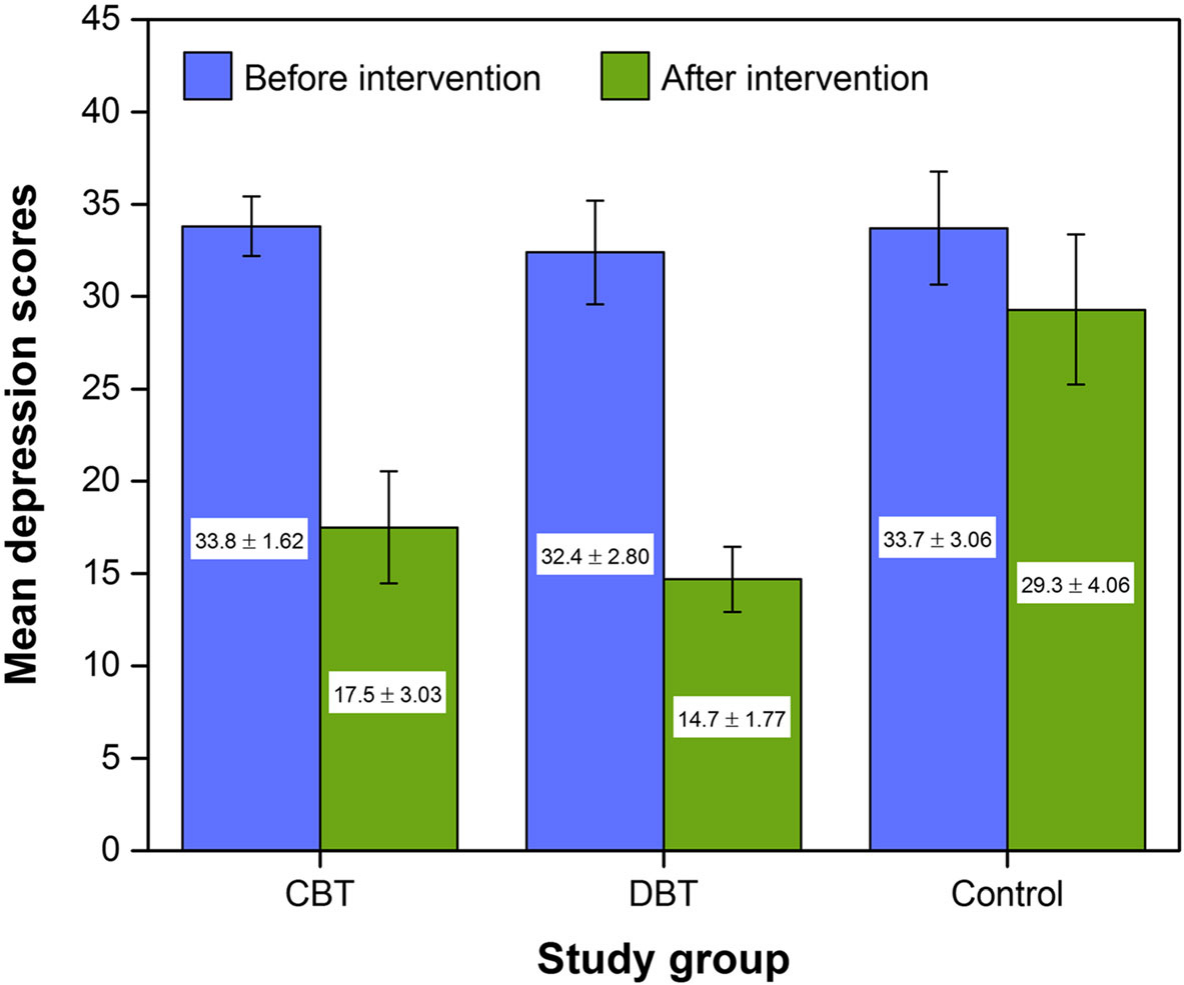
Mean of depression scores before and after intervention in the studied groups. Error bars display standard deviation (SD) of the mean

The mean and standard deviation of the obtained scores in the experimental and control groups are presented in Fig. 1. These values do not include any adjustments made by the use of a covariate in the analysis. A comparison of the mean depression score before intervention in different groups involving CBT, DBT, and control groups showed no significant difference. However after intervention, the mean of depression score decreased significantly in CBT and DBT groups as compared to the control group.

The Kolmogorov–Smirnov test was used to evaluate the assumption of normality of the variables. The results of this test (*Z* = 0.66, *p* value = 0.77) illustrate that according to the significance level, the variable of depression follows the assumption of normality (*p* > 0.05). Also, in order to establish the assumption of equality of variances, Levene’s *F* test should not be significant. The results of Levene’s *F* test (*F* = 0.26, *p* value = 0.62) show that the significance level for variable of depression is higher than 0.05. Consequently, the results of Kolmogorov–Smirnov and Levene’s *F* tests, suggest that the analysis of covariance (ANCOVA) test can be used to explore for differences in post-depression scores between the treatment groups with pre-depression scores treated as a covariate. Tables 3 and 4 show the results of the ANCOVA test, respectively, for the CBT and DBT treatment groups. Results indicate significant differences between baseline and post-intervention scores for depression (*F*[1,17] = 51.91, *p* value < 0.001 and *F*[1,17] = 106.62, *p* value < 0.001, respectively, for CBT and DBT groups). With respect to the effects of CBT and DBT skills training on reducing depressive symptoms, the effectiveness of these therapies was examined separately for the CBT (*η*_p_^2^, 0.75) and DBT skills training (*η*_p_^2^, 0.86) groups, compared with the control group. Cohen’s standard states that the effect size is small if *η*^2^ is 0.2. It is medium if *η*^2^ is 0.5, and it is large if *η*^2^ is 0.8 (Cohen, 1988; Lakens, 2013; Thompson, 2007). The obtained effect sizes in this study ranged from moderate to large, with highest effect size being found for the DBT intervention. The partial *η* values for CBT and DBT also showed that respectively almost 75 and 86% of the observed differences in depressive symptoms among women substance abusers after intervention were due to the effect of CBT and DBT training. Therefore, CBT and DBT trainings are capable of decreasing depression in substance abusers. However, no meaningful difference was found between CBT and DBT interventions regarding changes in the depression score after interventional training.

**Table 3:** Results of ANCOVA test for CBT treatment group

Source	Sum of squares	*df*	Mean square	*F*	*p* value	Partial eta squared (*η*_p_^2^)
Corrected model	699.59	2	349.79	26.17	< 0.001	0.76
Intercept	81.26	1	81.26	6.08	0.03	0.26
Pre-depression scores	3.39	1	3.39	0.25	0.62	0.02
Group	693.79	1	693.79	51.91	< 0.001	0.75
Error	227.21	17	13.37			
Total	11,878	20

**Table 4:** Results of ANCOVA test for DBT treatment group

Source	Sum of squares	*df*	Mean square	*F*	*p* value	Partial eta squared (*η*_p_^2^)
Corrected model	1074.14	2	537.07	54.39	< 0.001	0.87
Intercept	123.72	1	123.72	12.53	0.003	0.42
Pre-depression scores	8.34	1	8.34	0.84	0.37	0.05
Group	1052.78	1	1052.78	106.62	< 0.001	0.86
Error	167.86	17	9.87			
Total	10,922	20				

## Discussion

This study aimed at comparing the effectiveness of the cognitive and dialectical behavioral therapies (CBT, DBT) in reducing depressive symptoms in Iranian women substance abusers. Regarding the findings of this study, it can be concluded that the scores obtained in the depressive symptoms in the experimental groups had experienced a reduction as compared to the control group’s scores.

Beneficial effects of dialectical behavioral therapy (DBT) on depressive symptoms have been previously demonstrated in several studies. In a study by Harley, Sprich, Safren, Jacobo, and Fava (2008), a 16-session training program covering the four DBT skill sets of mindfulness, interpersonal effectiveness, emotion regulation, and distress tolerance led to pronounced improvements in depressive symptoms as compared to the control condition. In a study of depressed elderly patients who met the criteria for a personality disorder, investigators compared an adapted version of DBT plus antidepressant medications to medications only (Lynch et al., 2003). Findings indicated that a larger proportion of DBT patients were in remission from depression at post-treatment and at the 6-month follow-up period. In another study among older adults susceptible to major depression, a significant difference was found at the 6-month follow-up period, where 75% of the patients who were treated with antidepressant medication plus DBT were in remission as compared to only 31% of the medication-alone treatment group (Lynch et al., 2007). Although treatment effects of DBT on depression and personality disorder—especially in the elderly—were demonstrated previously, we could find few studies on the effects of this protocol on depressive symptoms in substance abusers. According to our analyses, by focusing on the effective role of DBT on reducing depressive components in substance abusers, it is apparent that this treatment has high effectiveness in lowering the severity of depression. In a similar study by Axelrod, Perepletchikova, Holtzman, and Sinha (2011) on women with substance dependence and borderline personality disorder (BPD) receiving dialectical behavioral therapy, this treatment approach was found to improve emotion regulation and mood and decrease substance use frequency. In another study by Courbasson, Nishikawa, and Dixon (2011) on substance use disorders (SUDs), results from the DBT condition revealed that the intervention had a significant positive effect on behavioral and attitudinal features of disordered eating, substance use severity and use, negative mood regulation, and depressive symptoms in the patients. Patients suffering from substance abuse have significantly higher behavioral, legal, and medical problems, including alcoholism and depression, and are more extensively involved in substance abuse if they also have a personality disorder. Thus, reducing their depression along with controlling their substance use can effectively result in curbing crime in the community. Our study also confirmed that cognitive behavioral therapy (CBT) has similar effects on depression in substance abusers as compared to dialectical behavior therapy (DBT). More evidences are available on the effects of CBT on relieving depressive symptoms in addicts. In a study by Curry, Wells, Lochmann, Craighead, and Nagy (2003) on depressed, substance-abusing adolescents, parent interviews demonstrated significant improvement in adolescent substance abuse even as adolescent measures demonstrated significant improvement in both the frequency of substance use and depressive symptoms. In a study by Watkins et al. (2012) carried over 3 months of treatment, those receiving both substance abuse treatment and CBT had mild symptoms of depression, as compared to moderate symptoms among those receiving only substance abuse treatment. About 55.8% of those undergoing combined treatment reported minimal symptoms, while 33.6% in the control group reported moderate symptoms.

The present study had some limitations mentioning which can pave the way for researchers to attempt to eliminate these limitations.

1. The sample of this study was just limited to the addiction treatment clinics in Birjand.
2. The participants placed in this study were all selected among female addict patients. Therefore, this makes it difficult to generalize the results to males.
3. Another limitation of this research was the existence of disruptive variables, such as the lack of suitable physical environment for intervention, the presence of secondary stressors, and the biological differences of individuals that may be accumulated randomly in a group. Since homogenization of depressive variables, such as depression severity and history of concomitant illness with depression or prior to depression, were not provided completely, caution should be exercised in interpreting and generalizing the results.
4. The small sample size is the main limitation of this study that increased the standard error of measurement and reduces our ability to generalize findings to a larger population. Therefore, additional longitudinal studies with larger samples are necessary to confirm our findings.

## Conclusions

In total, because of the importance of preventing depression and anxious moods, which lead to high rate of criminal behavior in women substance abusers, it is essential to deliver appropriate evidence-based mental health care in planning substance abuse programs by enhancing and expanding the clinical roles of substance abuse counselors. This is important because the lack of efficient treatment for depression in substance abusers impedes their recovery and can lead to relapse.

## Abbreviations

ANCOVA: Analysis of covariance
ANOVA: Analysis of variance
BDI: Beck Depression Inventory
CBT: Cognitive behavioral therapy
DBT: Dialectical behavioral therapy
DSM: Diagnostic and statistical manual of mental disorders
SD: Standard deviation
